# Happy in The Homeland? Satisfaction with Life and its Correlation with Flourishing and Affect Balance in Foreign-trained physicians who have Repatriated to Pakistan

**DOI:** 10.1192/j.eurpsy.2025.742

**Published:** 2025-08-26

**Authors:** M. Fatima, A. M. Hashmi, M. T. Farooq

**Affiliations:** 1 Psychiatry, Dublin North Mental Health Service, Dublin, Ireland; 2 Academic Department of Psychiatry and Behavioral Sciences, King Edward Medical University, Lahore, Pakistan; 3 Psychiatry, Gartnavel Royal Hospital, Glasgow, United Kingdom

## Abstract

**Introduction:**

International Medical Graduates add up to nearly one-third (23-28%) of the total physician workforce in the US, the UK, Australia, and Canada, of which around 40%-75% are from low-income countries. Pakistan is one of the three leading source countries following India and the Philippines. As per 2002 statistics by the Bureau of Emigration and Foreign Employment, only 10-15% of emigrated Pakistani physicians repatriate. No official data on the exact number of repatriated Pakistani physicians are available. This is the first original study that assessed satisfaction with life in physicians repatriating to a lower-middle-income country.

**Objectives:**

To assess satisfaction with life (SWL) and its correlation with psychological well-being in foreign-trained, repatriated Pakistani physicians.

**Methods:**

We conducted this cross-sectional study from April’22 to Nov’23, through purposive sampling among foreign-trained Pakistani physicians who repatriated at least three months before participating. We used the Scale of Positive and Negative Emotions (SPANE), Flourishing Scale, and Satisfaction with Life (SWL) scale. After transforming data to normality in SPSS 25 through the Distribution Fraction method, the independent sample t-test, and one-way ANOVA were applied. We assessed the correlation between affect balance, flourishing, and SWL through Pearson’s correlation and ascertained the predictors of SWL through binary logistic regression (α=0.05).

**Results:**

Of 109 respondents, the majority (70.6%) were males, from Punjab (83.5%), trained in USA/Canada (68.8%), and from the private sector (69.7%), with a Mean±SD age of 47.31±7.9. The total Mean ± SD SWL scores were 27.48± 5.03. 99 (90.8%) were satisfied with life while only 9.2% weren’t. The currently married respondents (27.8±4.9 vs 24.6±5.4, p=0.04) while those from Sindh and KPK provinces had lower scores. We found a positive moderate correlation between SWL and flourishing (r=0.488), positive emotions (r=0.391), and affect balance (r=0.327) all at p=<0.001. We found good fitness of the final model with SWL as the outcome variable and Flourishing and Overall Affect Balance as predictors: Omnibus Tests of Model Coefficients (p=0.001) and Hosmer and Lemeshow tests (p=0.322). Only flourishing predicted SWL and with higher perceived flourishing, there were 1.3x higher odds of SWL.

**Conclusions:**

This is the pioneer study to have addressed SWL and its correlation with psychological well-being in repatriated physicians, who using their skill sets and expertise, may help strengthen the healthcare system of the lower-middle income countries of origin, hence it’s imperative to identify factors linked to their psychological well-being. We recommend further research on this particularly qualitative exploration.

**Disclosure of Interest:**

None Declared

